# Traumatic Self-Inflicted Ventricular Laceration: A Case of Smith-Lemli-Opitz Syndrome in an Adult

**DOI:** 10.7759/cureus.53613

**Published:** 2024-02-05

**Authors:** Jennifer J Beuschel, Grace I Ng, Joanna C Abaraoha, Robert J Fortuna

**Affiliations:** 1 Internal Medicine and Pediatrics, University of Rochester Medical Center, Rochester, USA

**Keywords:** traumatic ventricular septal defect, childhood congenital conditions, intellectual and developmental disability, smith-lemli-opitz syndrome, self-injurious behavior, transition of care

## Abstract

Adults with intellectual and developmental disabilities (IDD) are increasingly living into adulthood, highlighting the need for adult clinicians to expand their familiarity with congenital conditions. Smith-Lemli-Opitz syndrome (SLOS) is a rare autosomal recessive inborn error of cholesterol synthesis. SLOS is commonly diagnosed in childhood, but a number of adults with IDD progress into adulthood without a formal diagnosis. We present an 18-year-old male with a history of IDD and altered pain sensation who was hospitalized following a self-inflicted knife injury resulting in a traumatic ventricular septal defect. Over the following 15 years, the patient continued to exhibit self-injurious behaviors. At the age of 33, caregivers consented to further work-up of his intellectual disability, and whole-exome genetic sequencing revealed a diagnosis of SLOS. The clinical course of this patient represents a unique presentation of altered pain sensation, a delayed diagnosis of SLOS into adulthood, and the challenges of providing care to an adult with IDD. The case further highlights the importance of understanding the typical workup and management of genetic and congenital conditions arising in childhood.

## Introduction

With ongoing scientific and medical advancements, more children with chronic conditions are living into adulthood. However, many young adults with childhood chronic conditions frequently encounter barriers to healthcare, including a lack of familiarity among adult clinicians and limitations of available diagnostic testing during the patient’s childhood [1,2]. As more adolescents and young adults age out of pediatric care, adult clinicians will increasingly need to care for patients with childhood-onset health conditions. One such condition is Smith-Lemli-Opitz syndrome (SLOS), a rare genetic syndrome first reported in Drs. Smith, Lemli, and Opitz’s 1964 paper describing three boys with similar congenital anomalies [3]. SLOS is characterized by mutations in 7-dehydrocholesterol reductase and may manifest through a broad range of intellectual and physical phenotypes [4]. Behavioral signs, such as poor feeding, and physical exam findings, such as syndactyly, often provide clues early in life, subsequently leading to a diagnosis. However, SLOS is occasionally not recognized until adulthood.

## Case presentation

An 18-year-old male with a history of developmental delay, intellectual disability with a reported IQ of 60, a cleft palate repaired in infancy, and a prior gastrostomy tube during childhood presented with a self-inflicted penetrating stab wound to his left anterior chest in the precordial area at the mid-clavicular line with a 3-inch paring knife. He had no reported history of depression, substance use, or any other psychiatric history. Due to the altered pain sensation, the patient was reportedly attempting to stimulate his heart with the paring knife. He was emergently taken to the operating room, where a 1-centimeter right ventricular laceration was identified and repaired. A post-operative echocardiogram demonstrated a mid-septal ventricular septal defect with moderate shunting. The patient returned to the operating room on post-op day seven for a ventricular septal defect repair.

He had a previous history of self-injurious behavior that had not required surgical intervention. His family history was notable for having two brothers and an aunt with intellectual disabilities. The patient lived at home with his parents and attended high school with an individualized education plan (IEP) and 1:1 support. He was active in the band and did not consume tobacco, alcohol, or other substances.

**Figure 1:**
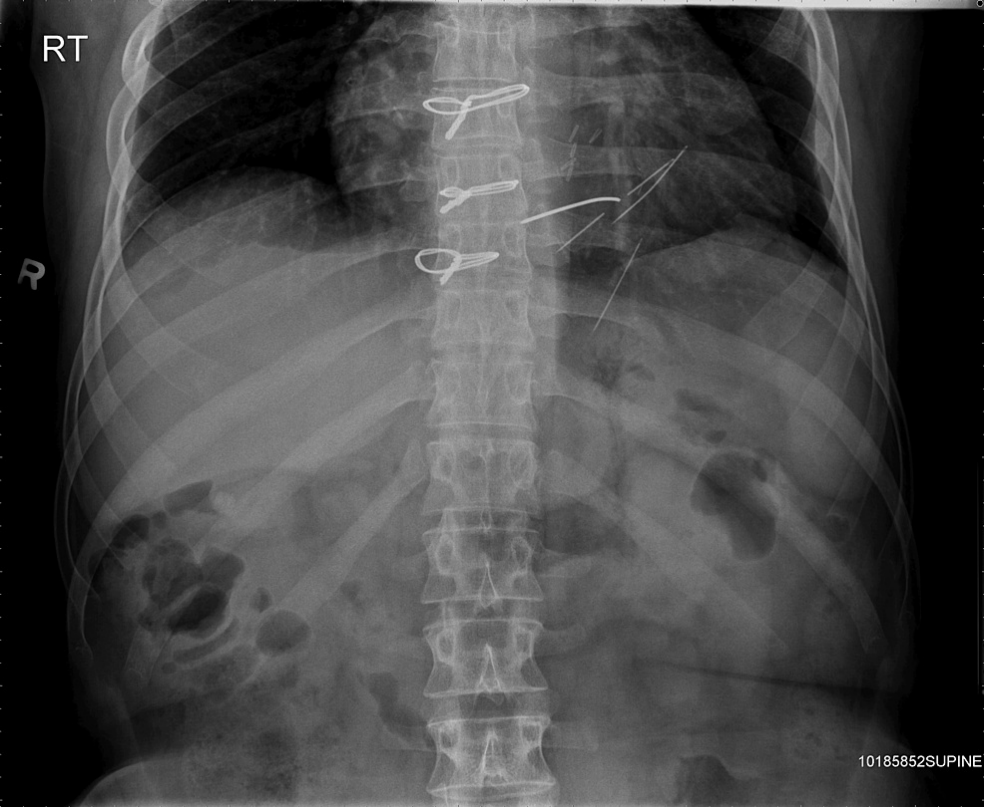
X-ray with a paperclip and sewing needle inserted in the pericardium and myocardium.

**Figure 2:**
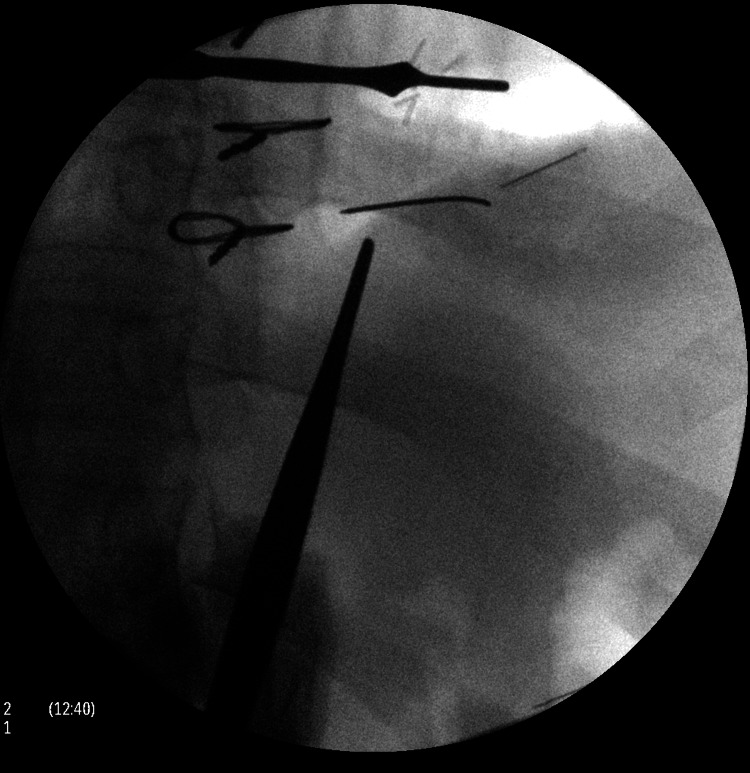
Surgical removal of the paperclip and sewing needle.

**Figure 3:**
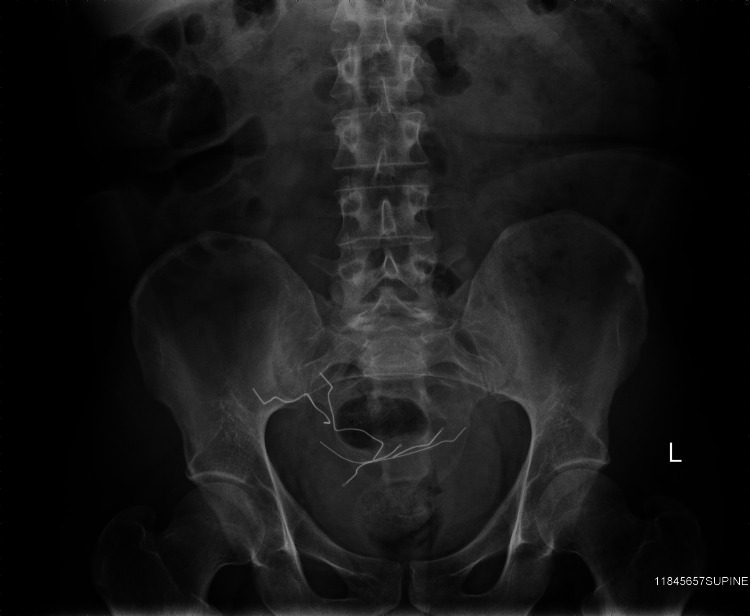
Abdominal X-ray with paperclips embedded in the pelvic floor.

**Figure 4:**
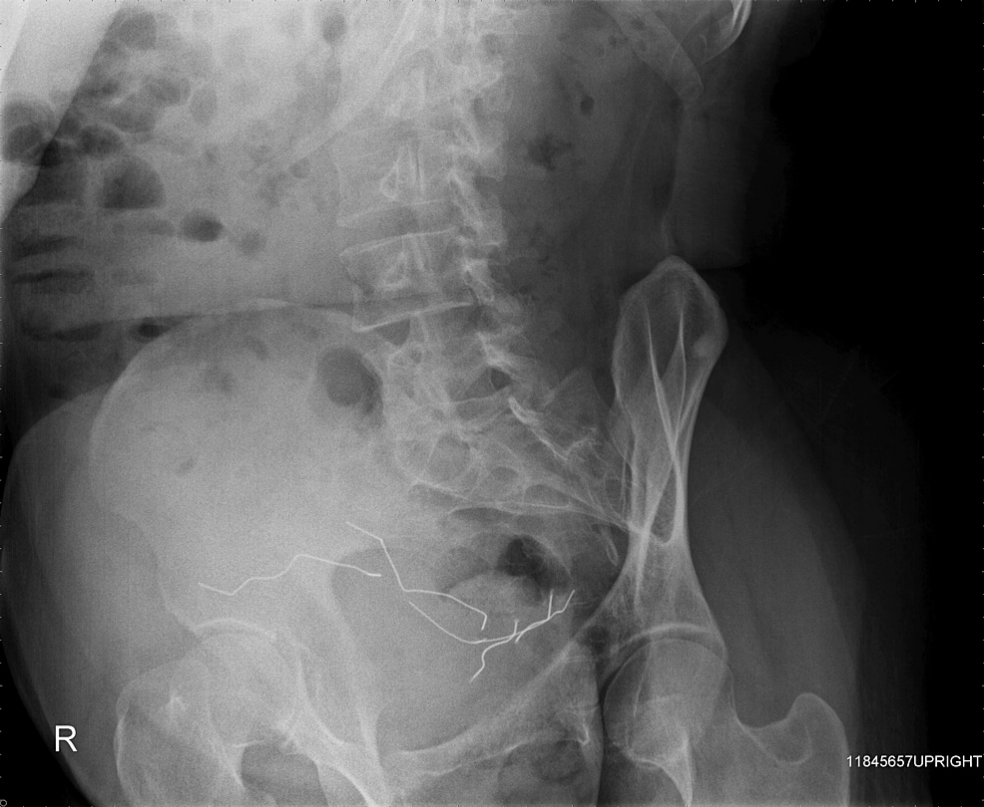
Lateral X-ray with paperclips embedded in the pelvic floor.

After the patient’s ventricular septal repair, the patient continued to attempt to stimulate his heart by inserting paperclips and sewing needles through his thoracic wall into his myocardium (Figure 1). He described no pain whatsoever, but enjoyed the resulting palpitations. The patient returned again to the operating room four years later, where multiple sewing needles and a paperclip were removed from his pericardium and myocardium (Figure 2). Over the following years, the patient began inserting paperclips into his abdominal cavity. The paperclips slowly migrated through his peritoneal cavity and became embedded within his pelvic floor musculature (Figures 3, 4). 

At the age of 33, caregivers consented to further work-up of his intellectual and congenital disability, and he subsequently underwent whole-exome sequencing. This revealed two heterozygous pathogenic variants in the DHCR7 gene, which is associated with SLOS. 

Placement and long-term living arrangements presented a significant challenge. After a seven-month hospitalization for safety, he was transferred to a group home with 24-hour line-of-site supervision. He has been managed medically and remains stable without further self-inflicted injuries or worsening cardiac function. 

## Discussion

The clinical course of this patient highlights an unusual presentation of altered pain sensation in Smith-Lemli-Opitz syndrome, delayed diagnosis into adulthood of a congenital condition, and the challenges of providing care to an individual with a severe intellectual disability. There were many factors that contributed to the delay in diagnosis. During the patient’s childhood, whole-exome genetic sequencing was not available. In addition, ethical concerns related to genetic testing were raised by the caregivers, further delaying the diagnosis. Although many patients carry a diagnosis of IDD as they enter adulthood, many, in fact, have an undiagnosed genetic condition. With the availability of new diagnostic tests, more formal diagnoses may provide additional therapeutic benefit to patients and guide their life care. 

**Figure 5:**
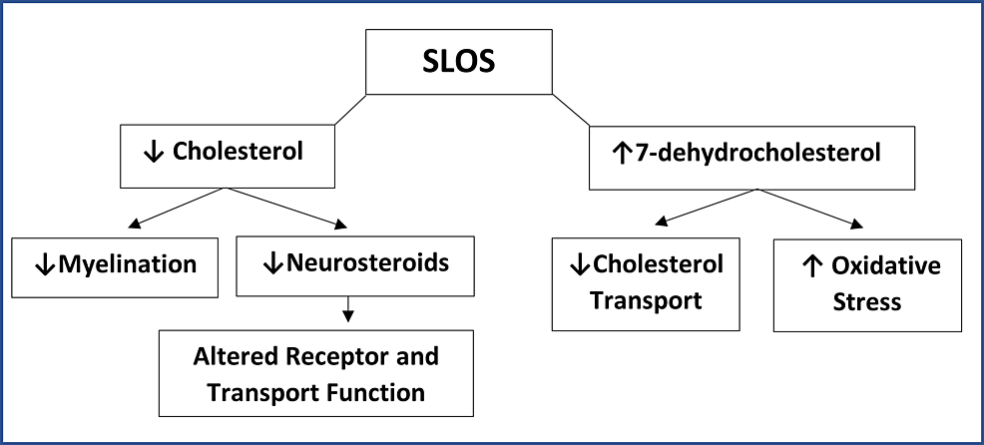
Impact of inborn errors in cholesterol synthesis (Smith-Lemli-Opitz syndrome). Adapted from "Pathogenesis SLOS", published under a Creative Commons License [7].

*Smith-Lemli-Opitz syndrome*: The patient was diagnosed with Smith-Lemli-Opitz syndrome at the age of 33 by way of whole exome sequencing. In brief, Smith-Lemli-Opitz syndrome is an autosomal recessive, multisystem syndrome caused by a deficiency of 7-dehydrocholesterol reductase, which is needed for proper cholesterol metabolism (Figure 5) [5]. The manifestations of this condition can range from mild to severe. Given the brain’s relatively higher concentration of cholesterol, neurological and behavioral manifestations are commonly seen in SLOS [5]. In the case of our patient, he had a history of developmental delay and intellectual disability throughout life (delayed gross motor skills and speech), as well as self-injurious behavior since the age of 17. While the specific pathophysiology of the self-injurious behavior and pain pathways have yet to be elucidated in SLOS, self-inflicted injury is a documented manifestation of the condition, which, for our patient, resulted in a traumatic VSD [6].

*Genetic work-up for congenital conditions*: With medical advances and the expanding availability of genetic testing, such as whole genome or whole exome sequencing, the medical community is now able to recognize and diagnose conditions that were previously attributed to an idiopathic or unknown etiology [8]. Like many adults with rare conditions and IDD, our patient had symptoms since childhood, but a formal diagnosis was not made until the age of 33, after he underwent whole-exome sequencing. 

Familiarity with genetic testing is an important skill for clinicians caring for adults with conditions arising in childhood. For example, chromosomal microarray (CMA) analysis is considered the first-line genetic test for intellectual disabilities, replacing older modalities such as karyotyping and fluorescence in situ hybridization (FISH) (Table 1) [9,10]. In addition to IDD, genetic testing should also be considered to evaluate patients with conditions with known genetic associations, such as idiopathic cardiomyopathy, epilepsy, and certain types of chronic kidney disease [10-14].  

**Table 1:** Selected genetic testing and targets [15]

Testing Modality	Description	Clinical Use
Karyotype	Detects abnormalities in the count of chromosomes	Used to detect aneuploid conditions where the number of chromosomes is more or less at 46 Example: Trisomy 21
Chromosomal Microarray Analysis (CMA)	Uses probes to bind to specific chromosome regions and compare to a reference	Used to detect clinically significant microdeletions or duplications Frequently used in work-up of intellectual disability and global developmental delay
Genetic Sequencing	Targeted gene/molecular testing sequencing	Used to analyze for a specific mutation
	Gene panel testing	Analyzes regions in selected genes that are known to be associated with the presenting phenotype
	Whole exome sequence (WES)	Examines all coding regions of the genome
	Whole genome sequencing (WGS)	Examines the entire genome (coding and non-coding)

With the increase in genetic testing, there is a corresponding increase in the detection of variants of uncertain significance (VUS). For genetic results that do not yield a clear diagnosis, it is important to seek the assistance of a clinical geneticist and genetic counselor. Geneticists are available at many academic medical centers, and some have created an eConsult program for primary care physicians [16]. 

*Resources for patients with IDD*: Patients with IDD and congenital disability often experience multiple systemic and social challenges throughout childhood, highlighting the importance of both early identification and early intervention [17]. With health care advances, vulnerable populations, such as those with IDD, now have life expectancies approaching those of the general population. Entering adulthood with IDD and a congenital disability, however, can present a range of challenges for adult clinicians who might be unfamiliar with some congenital conditions. Older adults with IDD are at increased risk of experiencing poor health outcomes, emphasizing the need to increase clinician comfort with IDD and congenital conditions. In addition to gaining familiarity with condition-specific recommendations, adult clinicians should avoid the pitfall of not providing age-appropriate health maintenance. Adult clinicians should also become familiar with resources available for their patients with IDD related to housing, education, and job training (Table 2). For example, the state of New York manages the State Office for People with Developmental Disabilities (OPWDD), and other states have similar programs. Given the many challenges, it is important for adult clinicians to ensure that patients are linked to appropriate services as soon as possible.

**Table 2:** Examples of resources for patients with IDD and congenital disabilities [18-20] IDD: Intellectual and developmental disabilities

Resource	Description
National Resources	Disabled Peoples’ International (DPI) (https://disabledpeoplesinternational.org/)
State-Based Resources	NY Office for People with Developmental Disabilities (OPWDD) (https://opwdd.ny.gov/)
Condition-Based Resources	Smith-Lemli-Opitz Foundation (www.smithlemliopitz.org)

## Conclusions

As more young adults age out of pediatric care, adult clinicians will be increasingly caring for patients with childhood-onset health conditions. This case of Smith-Lemli-Opitz syndrome (SLOS) highlights a rare presentation of an autosomal recessive inborn error of cholesterol metabolism. The case further highlights the challenges it poses to the adult health care system and emphasizes the need for adult clinicians to develop comfort with the diagnosis of IDD and other congenital conditions. With advances in genetic testing, our case also demonstrates the importance of understanding the typical workup and management of genetic conditions. Finally, clinicians should familiarize themselves with available local and national resources that provide support for patients with IDD and their families.
